# Direct Visualization of the Two-step Nucleation Model by Fluorescence Color Changes during Evaporative Crystallization from Solution

**DOI:** 10.1038/srep22918

**Published:** 2016-03-08

**Authors:** Fuyuki Ito, Yukino Suzuki, Jun-ichi Fujimori, Takehiro Sagawa, Mitsuo Hara, Takahiro Seki, Ryohei Yasukuni, Marc Lamy de la Chapelle

**Affiliations:** 1 Department of Chemistry, Institute of Education, Shinshu University, 6-ro Nishinagano, Nagano 380-8544, Japan; 2 Department of Molecular Design and Engineering, Graduate School of Engineering, Nagoya University, Furo-cho, Chikusa, Nagoya 464-8603, Japan; 3 Université Paris 13, Sorbonne Paris Cité, Laboratoire CSPBAT, CNRS, (UMR 7244), 74 rue Marcel Cachin, F-93017 Bobigny, France

## Abstract

The two-step nucleation model for crystal nuclei formation explains several experimental and theoretical results better than the classical nucleation theory. We report here direct visualization of the two-step nucleation model for organic molecular crystallization. Evaporative crystallization from a solution of a dibenzoylmethane boron complex that displays mechanofluorochromism, a fluorescence color change induced by mechanical perturbation, was probed by fluorescence change. The dependence of fluorescence change on dispersion concentration of the complex in a polymer matrix was also investigated. We detected transitional emission from the amorphous cluster state prior to crystallization. This is the first demonstration of the two-step nucleation model based on fluorescence color changes.

Crystal formation from solution is essential in fundamental science as well as the fabrication of pharmaceuticals, food, polymers and organic solid materials, but remains poorly understood^1^,^2^,^3^. In solution crystallization, the formation of crystal nuclei plays an important role in determining the crystal structure, size and polymorph, thus controlling crystallization and crystal quality. In classical nucleation theory, molecules add on one by one to extend the crystal lattice and form an embryonic nucleus in a one-step process. The classical model for crystallization visualizes the formation of a metastable crystalline nucleus that reaches a critical size through density fluctuations and grows into a stable crystal. Some computational and experimental results, however, cannot be explained based only on classical nucleation theory^4^. Recently, the two-step nucleation model involving a liquid-like cluster intermediate prior to nucleation has been developed to explain protein crystallization, and has been shown to be of more general validity^1^. It is postulated that liquid-like clusters originate from disordered liquid or amorphous metastable clusters in homogeneous solutions^5^.

The fluorescence spectra of materials are sensitive to molecular environment and aggregation. In principle, fluorescence spectroscopy can be used to probe the progress of molecular assembly on the scale of just a few molecules or that of a bulk process. We investigated the fluorescence spectral change of perylene and a cyanostilbene derivative displaying aggregation-induced emission during evaporation by fluorescence microscopy^6^,^7^. Studying concentration-dependent fluorescence spectral changes of organic materials both in polymer films and during solvent evaporation provides information about molecular assembly and the nucleation and growth of crystals. Recently, Liu *et al*.^8^ monitored an amorphous-to-crystalline transformation process by fluorescence color changes.

In this paper, we investigate the fluorescence properties of 4,4′-di-*tert*-butyldibenzoylmethanatoboron difluoride (BF_2_DBMb, Fig. 1a) in poly(methyl methacrylate) (PMMA) films and solution during evaporative crystallization to visualize the two-step nucleation model. BF_2_DBMb exhibits mechanofluorochromism^9^,^10^, which originates from the different emission properties of its amorphous and crystalline states^11^. Fluorescence spectroscopy is used to identify the two-step nucleation model by detection of an amorphous state prior to crystallization by tracking fluorescence color changes of BF_2_DBMb.

First, we examined the fluorescence of BF_2_DBMb in dilute solution, crystals and amorphous state, as shown in Fig. 1b–d, respectively. The fluorescence is purple, blue and greenish orange for the dilute solution, crystal and amorphous state, respectively. The absorption and fluorescence spectra of BF_2_DBMb in 1,2-dichloroethane are presented in Fig. 1e. Absorption peaks were observed at 350, 370, and 390 nm; a mirror image of the fluorescence spectrum with peaks at 413 and 430 nm and a shoulder at 460 nm, which can be assigned to vibrational modes of the BF_2_DBMb monomer. The crystals exhibited fluorescence peaks around 445 and 470 nm, and the amorphous state around 550 nm (Fig. 1f). The crystalline and amorphous states were confirmed by X-ray diffraction (XRD) measurements (Figure S1). These findings indicate that the molecular forms of BF_2_DBMb such as monomer (isolated state) and aggregated states can be distinguished by fluorescence color changes.

The evolution of molecular assembly of BF_2_DBMb in a polymer matrix can be probed by fluorescence changes, which can provide information about aggregation and segregation processes. To confirm the molecular assembly process of BF_2_DBMb in a PMMA matrix by static measurements, the concentration dependence of the fluorescence changes was determined, as shown in Fig. 2. The close-up fluorescence image (ca. 22 × 22 mm) is uniformly blue for the PMMA film doped with 0.01 mol% BF_2_DBMb. With increasing concentration to 3.0 mol%, the fluorescence color changes to greenish-orange via light blue. Needles with sky-blue emission were observed in the film with 4.0 mol% BF_2_DBMb, which probably originate from the segregation of BF_2_DBMb crystals in the polymer film. At a lower concentration of 0.01 mol% BF_2_DBMb, fluorescence originating from the monomer was observed around 430 nm (Fig. 1e). The fluorescence peak red-shifted as BF_2_DBMb concentration increased to 0.5 mol%. For the film with 1.0 mol% BF_2_DBMb, the fluorescence peak was located at 450 nm and a broad shoulder appeared around 530 nm. The intensity of the broad emission band around 530 nm increased with BF_2_DBMb concentration up to 3.0 mol%, then it suddenly disappeared and the fluorescence peak shifted to around 450 nm. The fluorescence spectrum of the film with 4.0 mol% BF_2_DBMb is identical to that of the emission from a BF_2_DBMb crystal (see Fig. 1f). These results suggest that the molecular assembly process can be partially isolated or frozen by the polymer matrix to reveal the dynamics of crystal formation from a monomer state via an amorphous state of BF_2_DBMb consecutively^12^. These findings are reasonably supported by the XRD results obtained for polymer films (Figure S2). The fluorescence excitation spectra of BF_2_DBMb in PMMA films indicate exciton splitting of the band, suggesting the formation of H- and J-aggregates (Davydov splitting) with increasing BF_2_DBMb concentration (Figure S3). Based on the band splitting, the average number of molecules in each aggregate was estimated to be 12 (see the SI for details).

Next, we measured the fluorescence changes of BF_2_DBMb during evaporative crystallization from solution to detect the molecular assembly process. Figure 3a shows fluorescence images obtained during solvent evaporation from a droplet of 3.1 × 10^−2^ mol·dm^−3^ BF_2_DBMb in 1,2-dichloroethane, the movie of which is shown in the SI. At 0 s, the fluorescence of the droplet was purple. The emission color changed to orange from the edge of the droplet after 25 s. The emission of the whole droplet was orange around 32 s. Solvent evaporation caused the orange emission to form a doughnut-like shape around 34 s. From 34 s, the region of purple emission shrunk from both in- and outside the droplet. Finally, most of the droplet exhibited blue emission with small remaining regions with orange emission. The evaporation of solvent from the inner region of the droplet probably originated from an analogous mechanism to gas bubbles, which has been observed for a molecular assembly during evaporation of solvent with low vapor pressure^13^. Orange emission is not observed even at high concentration; it was exhibited only from a supersaturated solution. This strongly suggests that the molecular state or assembly with orange emission can only exist in solution in a non-equilibrium state.

To obtain spectroscopic information about the solvent evaporation process, we observed the fluorescence spectra of BF_2_DBMb in 1,2-dichloroethane during solvent evaporation as a function of time (Fig. 3b). The initial fluorescence spectrum of the droplet exhibits a peak at 433 nm, with shoulders at 415, 460, and 550 nm, corresponding to the emission spectrum of the monomer state. The intensity of the peak around 550 nm originating from the amorphous state increased monotonically over time until 91 s. The intensity of the fluorescence peak at 433 nm decreased from 91 to 95 s. After 95 s, peaks around 445 and 470 nm appeared concomitant with a decrease of the intensity of the 550-nm band. The series of fluorescence spectral changes corresponds to the changes of fluorescence images.

Based on the fluorescence properties of BF_2_DBMb described above, we can explain the molecular assembly process during solvent-evaporative crystallization. Crystals of BF_2_DBMb formed from solution via an amorphous state. The fluorescence changes are identical to the behavior observed as the concentration of BF_2_DBMb in PMMA films was increased. The Raman spectra of each state (Figure S4) are slightly different, strongly supporting the changes of molecular interactions in the ground state.

The fluorescence spectra were analyzed by nonlinear least-squares fitting with six Gaussians with peak position and width as listed in Table 1. Examples of the simulated spectra are provided in Figure S5. All observed spectra were reproduced well with these values. The positions of peak 1, 2, and 4 correspond to the monomer fluorescence band, those of peak 3 and 5 to the crystal, and that of peak 6 to amorphous emission. We plotted the relative abundance of monomer (peak 1, 2, 4), crystal (peak 3, 5) and amorphous (peak 6) states as a function of time, as shown in Fig. 4a. Immediately after drop formation, the fraction of monomer species was about 0.9. The monomer fraction decreased monotonically with increasing amorphous fraction up to 95 s. The amorphous fraction reached about 0.6 at 95 s, and then decreased considerably. No crystal fraction was observed before 95 s. The crystal fraction suddenly increased after 95 s concomitant with the decrease of the amorphous fraction. These findings indicate that crystals of BF_2_DBMb can be formed from monomer species via an amorphous state, which are presumed to show hierarchical change like a consecutive reaction, as schematically illustrated in Fig. 4b.

The fluorescence changes of BF_2_DBMb observed in PMMA films and during solvent-evaporative crystallization allowed direct visualization of the two-step nucleation model. The fluorescence color change from purple to blue via orange corresponds to the change in molecular formation from monomer to crystalline via an amorphous state. A transient amorphous state is formed prior to crystal formation. The two-step nucleation model suggests that a liquid-like cluster acts as a crystal nucleus, which has been established based on the induction time of a crystal formation rate^14^, NMR spectroscopy^15^,^16^, electron microscopy^17^, and nonphotochemical laser-induced crystallization^18^. In the present case, the observed orange emission originating from an amorphous species demonstrated the existence of a liquid-like cluster before crystallization. This amorphous state was only observed in the supersaturated region during solvent evaporation; i.e., the non-equilibrium state. The time evolution of the relative abundance of the molecular states of BF_2_DBMb clearly revealed that the amorphous species acted as a precursor for crystal formation. The results of fluorescence visualization during solvent-evaporative crystallization corroborate the two-step model for crystal formation^1^,^2^.

This study has clearly confirmed the two-step nucleation model by measuring fluorescence color changes. An intermediate state such as a liquid-like cluster has an important role in polymorph expression. The present method allows crystal formation to be observed using a conventional optical detection system under ambient conditions, making it attractive to study the control of polymorphism of organic emissive materials with multiple emissive states or colors depending on their phase, such as mechanofluorochromic materials. This method can also be used to directly visualize Ostwald’s rule of stages during the phase change of organic molecular solids; this study is in progress.

## Method

BF_2_DBMb was prepared by standard Claisen condensation, and the boronation was performed using BF_3_∙OEt_2_ in CH_2_Cl_2_ according to a previous report^11^. BF_2_DBMb was purified by column chromatography on silica gel (hexane/ethyl acetate = 5:1) and recrystallization from acetone. ^1^H NMR (CDCl_3_) δ 8.10 (d, 4 H, *J* = 9.0 Hz, ArH), 7.58 (d, 4 H, *J* = 9.0 Hz, ArH), 7.14 (s, 1 H, COCHCO), 1.37 (s, 18 H, (CH_3_)_3_C-Ar). M.p. 272 °C.

PMMA with a number-average molecular weight (M_n_) of 3.5 × 10^5^ was purchased from Aldrich and used as received. BF_2_DBMb-doped PMMA thin films were prepared by drop-casting 1,2-dichloroethane solutions with a fixed PMMA concentration of 2 wt% onto quartz plates. The cast films were dried under vacuum for 24 h at 298 K. The films for XRD measurements were prepared by the following procedure. PMMA was dissolved in CH_2_Cl_2_ (Kanto Chemical Co., Inc.) to give a PMMA concentration of 2 wt%. BF_2_DBMb was added to give a concentration of 1–4 mol% relative to MMA units in the CH_2_Cl_2_ solution. BF_2_DBMb/PMMA hybrid films were prepared by spin-coating the mixtures on glass substrates at 500 rpm for 1 min. Flakes of the films were added to polyimide cells composed of Upilex^®^-7.5 S (Ube Industries, Ltd.).

Fluorescence spectra were recorded with a Shimadzu RF-5300PC fluorescence spectrophotometer at an excitation wavelength of 370 nm. Fluorescence color and intensity, as well as the morphology of the crystals, were captured with an inverted fluorescence microscope (Olympus IX71) equipped with a UPlanFl 4 × /0.13 PhL (Olympus) objective lens, and CCD camera (Sigma Koki SK-TC202USB-AT). The solvent evaporation process was observed by a USB 4000 (Ocean Optics) spectrometer. A light-emitting diode (Siokaze Engineering UVF-365 AC, *λ* = 365 nm, 0.03 mW) was used as the excitation source. A 10-μL droplet of a 1,2-dichloromethane solution of 3.1 × 10^−2^ mol·dm^−3^ BF_2_DBMb was dropped onto a cover slip. A movie of the droplet during solvent evaporation was captured by a conventional digital camera (Canon PC1251), and was not corrected for the wavelength dependence of the detectors. In this measurement, the time origin was after the charge-coupled device camera was focused. This is the reason for the indicated time difference between the movie and spectral changes described below. Raman spectra were measured using a confocal Raman microscope (XploRA, Horiba Jobin–Yvon) and excitation wavelength of 660 nm. Powder XRD patterns of crystalline and amorphous states were measured by a Rigaku MiniFlex600 diffractometer. The XRD patterns of films were obtained with an FR-E diffractometer (Rigaku) detected by an R-axis IV two-dimensional detector (Rigaku) using an imaging plate. The samples in the polyimide cells were measured by irradiation with an X-ray beam for 10 min. All experiments were performed at room temperature.

## Additional Information

**How to cite this article**: Ito, F. *et al*. Direct Visualization of the Two-step Nucleation Model by Fluorescence Color Changes during Evaporative Crystallization from Solution. *Sci. Rep.* **6**, 22918; doi: 10.1038/srep22918 (2016).

## Supplementary Material

Supplementary Movie 1

Supplementary Information

## Figures and Tables

**Figure 1:**
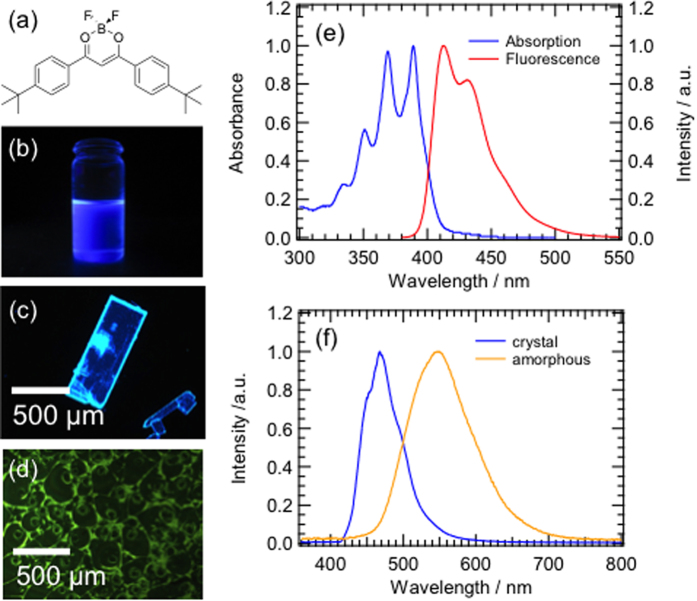
(**a**) Molecular structure of BF_2_DBMb. Fluorescence images of BF_2_DBMb in (**b**) 1,2-dichloromethane, (**c**) crystalline state, and (**d**) amorphous state under 365-nm UV irradiation. (**e**) Absorption and fluorescence spectra of BF_2_DBMb, and (**f**) fluorescence spectra of crystal and amorphous states of BF_2_DBMb following excitation at 380 nm.

**Figure 2:**
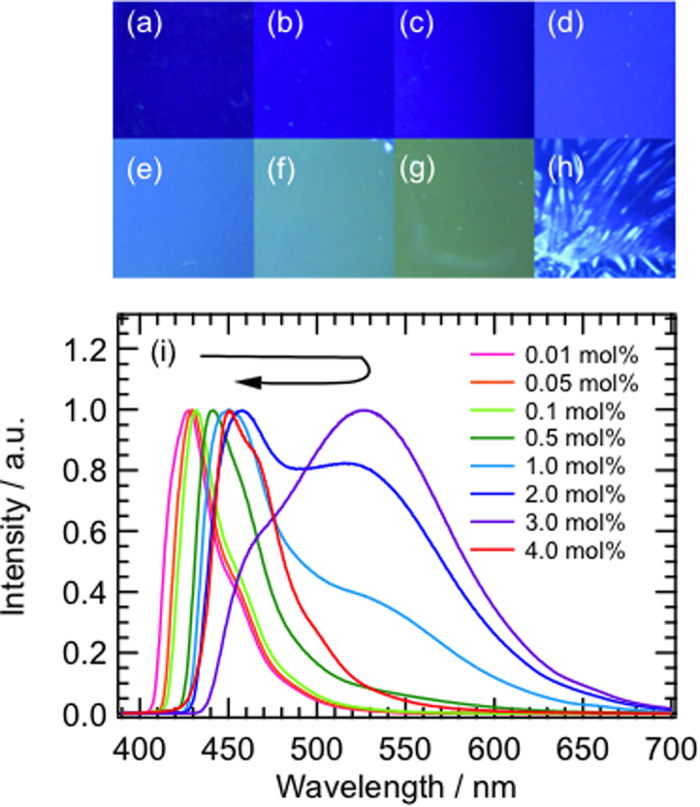
Close-up fluorescence images of BF_2_DBMb in PMMA films with a concentration of (**a**) 0.01, (**b**) 0.05, (**c**) 0.1, (**d**) 0.5, (**e**) 1, (**f**) 2, (**g**) 3, and (**h**) 4 mol%, and (**i**) fluorescence spectra as a function of concentration obtained by excitation at 380 nm.

**Figure 3:**
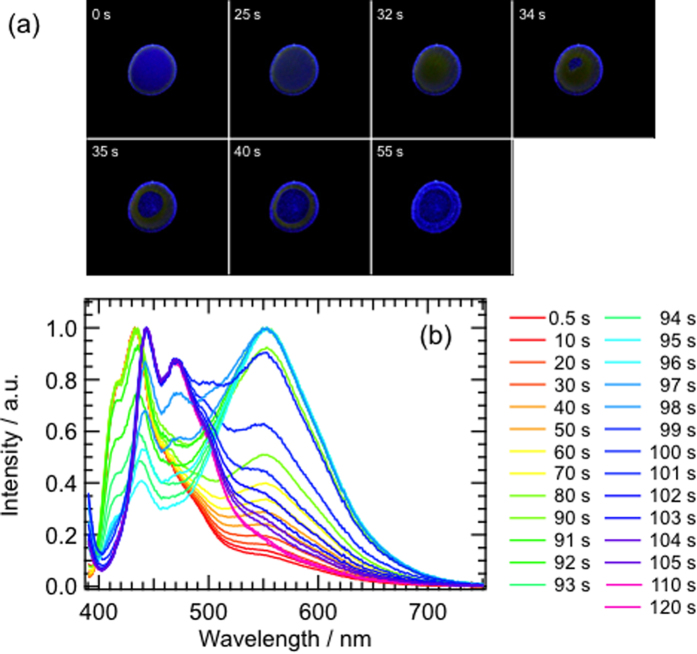
(**a**) Fluorescence images of a droplet of BF_2_DBMb in 1,2-dichloroethane during evaporation under 365-nm UV irradiation. The droplet diameter is about 5 mm. (**b**) Changes of fluorescence spectra of BF_2_DBMb in 1,2-dichloroethane during solvent evaporation.

**Figure 4:**
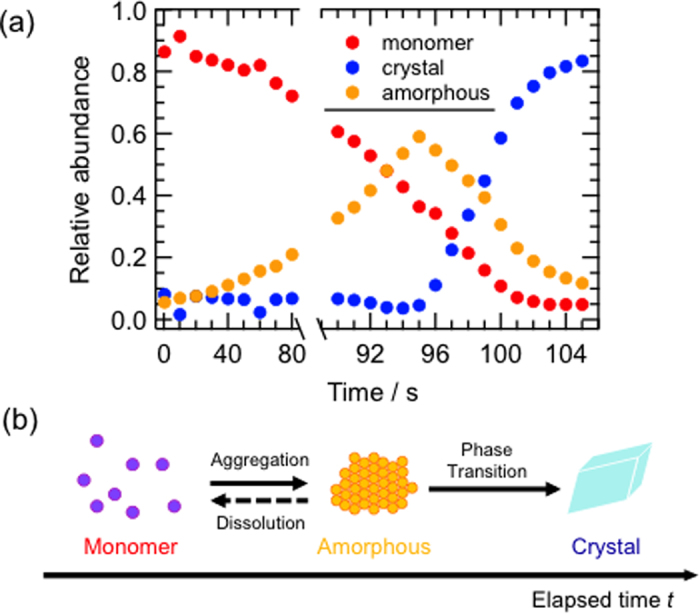
(**a**) Change of the relative abundance of monomer, amorphous and crystal states based on time-resolved fluorescence spectral measurements. (**b**) Schematic representation of the molecular assembly process based on the changes of fluorescence spectra.

**Table 1:** Position and width of Gaussians peaks used to simulate the fluorescence spectral changes of BF_2_DBMb.

	Peak 1	Peak 2	Peak 3	Peak 4	Peak 5	Peak 6
Position/cm^−1^	24237	23124	22700	21854	21380	18150
(/nm)	(413)	(432)	(441)	(458)	(468)	(551)
Width/cm^−1^	680	700	540	1650	1930	2000
