# Supplementary material for: Direct Visualization of the Two-step Nucleation Model by Fluorescence Color Changes during Evaporative Crystallization from Solution
Source: Sci Rep. 2016 Mar 8;6:22918. doi: 10.1038/srep22918 (PMC4782171; doi:10.1038/srep22918)
Supplement: Supplementary Information [file srep22918-s2.doc]

Supplementary Information for

Direct Visualization of the Two-step Nucleation Model by Fluorescence Color Changes during Evaporative Crystallization from Solution

Fuyuki Ito,^*,†^ Yukino Suzuki,^†^ Jun-ichi Fujimori,^†^ Takehiro Sagawa,^†^ Mitsuo Hara,^‡^ Takahiro Seki,^‡^ Ryohei Yasukuni,^¶^ and Marc Lamy de la Chapelle^¶^

^†^Department of Chemistry, Institute of Education, Shinshu University, 6-ro, Nishinagano, Nagano 380-8544, Japan

^‡^Department of Molecular Design and Engineering, Graduate School of Engineering, Nagoya University, Furo-cho, Chikusa, Nagoya 464-8603, Japan

^¶^Université Paris 13, Sorbonne Paris Cité, Laboratoire CSPBAT, CNRS, (UMR 7244), 74 rue Marcel Cachin, F-93017 Bobigny, France

**Figure S1**. XRD patterns for BF_2_DBMb in crystalline form (blue line) and an amorphous state after melting (orange line). The sharp peak at 30° for the amorphous sample is an artifact of the measurements.

**Figure S2**. XRD patterns of BF_2_DBMb/PMMA films in a polyimide cell as a function of BF_2_DBMb concentration.

**Figure S3**. (a) Fluorescence excitation spectra of BF_2_DBMb in a PMMA matrix monitored at 440 nm. (b) The fluorescence peak shift (orange) and estimated number of molecules in each aggregate (blue) in PMMA films as a function of BF_2_DBMb concentration.

Figure S3a shows the fluorescence excitation spectra of BF_2_DBMb in a PMMA matrix as a function of BF_2_DBMb concentration. Peaks of the excitation spectra split as the BF_2_DBMb concentration increased. The transition energy and number of molecules in one-dimensional aggregates are related as follows:[^1^](#_ENREF_1)

*E*_N_ = *E*_1_–((*N*–1)/*N*)(*E*_1_–*E*_∞_), (1)

where *E*_N_ is the transition energy of an aggregate consisting of *N* molecules, *E*_1_ is the transition energy of an isolated molecule, and *E*_∞_ is the transition energy of an infinite chain. We roughly estimated the number of molecules in each aggregate formed in the PMMA films by assuming that *E*_1_ and *E*_∞_ were 25510 and 24546 cm^-1^, respectively. The calculated results are plotted as blue circles in Figure S3b.

**Figure S4**. Raman spectra of BF2DBMb in a concentrated solution with orange emission monitored during solvent evaporation (orange line), dilute solution with purple emission (purple line), amorphous state after melting (red line), a single crystal (blue line) and the Raman spectrum of 1,2-dichloroethane (black dashed line).

The Raman spectra of BF2DBMb in different states are shown in Figure S4. There are large contributions from the solvent at 705, 741, 1162 and 1428 cm^-1^ in the spectra obtained in solution. The spectrum obtained for the concentrated solution is different from those obtained under other conditions. This result strongly supports the formation of transient intermediates during evaporative crystallization.

**Figure S5**. Resolved fluorescence spectra of BF_2_DBMb during solvent evaporation fitted by six Gaussians.

**References**

(1) Hsu, Y.; Penner, T. L.; Whitten, D. G*. J. Phys. Che*m**. 19**92*,* 96, 2790.
